# The Severe Typhoid Fever in Africa Program: Study Design and Methodology to Assess Disease Severity, Host Immunity, and Carriage Associated With Invasive Salmonellosis

**DOI:** 10.1093/cid/ciz715

**Published:** 2019-10-30

**Authors:** Se Eun Park, Trevor Toy, Ligia Maria Cruz Espinoza, Ursula Panzner, Ondari D Mogeni, Justin Im, Nimesh Poudyal, Gi Deok Pak, Hyeongwon Seo, Yun Chon, Heidi Schütt-Gerowitt, Vittal Mogasale, Enusa Ramani, Ayan Dey, Ju Yeong Park, Jong-Hoon Kim, Hye Jin Seo, Hyon Jin Jeon, Andrea Haselbeck, Keriann Conway Roy, William MacWright, Yaw Adu-Sarkodie, Ellis Owusu-Dabo, Isaac Osei, Michael Owusu, Raphaël Rakotozandrindrainy, Abdramane Bassiahi Soura, Leon Parfait Kabore, Mekonnen Teferi, Iruka N Okeke, Aderemi Kehinde, Oluwafemi Popoola, Jan Jacobs, Octavie Lunguya Metila, Christian G Meyer, John A Crump, Sean Elias, Calman A Maclennan, Christopher M Parry, Stephen Baker, Eric D Mintz, Robert F Breiman, John D Clemens, Florian Marks

**Affiliations:** 1 International Vaccine Institute, Seoul National University Research Park, Republic of Korea; 2 Oxford University Clinical Research Unit, Ho Chi Minh City, Vietnam; 3 Department of Microbiology and Infectious Disease, B. P. Koirala Institute of Health Sciences, Dharan, Nepal; 4 Institute of Medical Microbiology, University of Cologne, Germany; 5 Department of Medicine, Cambridge University, United Kingdom; 6 Global Health Institute, Emory University, Atlanta, Georgia; 7 School of Public Health, and, Kwame Nkrumah University of Science and Technology, Kumasi, Ghana; 8 Kumasi Centre for Collaborative Research in Tropical Medicine, Kwame Nkrumah University of Science and Technology, Kumasi, Ghana; 9 University of Antananarivo, Madagascar; 10 Institut Supérieur des Sciences de la Population, University of Ouagadougou, Ouagadougou, Burkina Faso; 11 Schiphra Hospital, Ouagadougou, Burkina Faso; 12 Armauer Hansen Research Institute, ALERT Campus, Addis Ababa, Ethiopia; 13 Faculty of Pharmacy, University of Ibadan; 14 Department of Medical Microbiology and Parasitology, College of Medicine, University of Ibadan; 15 Department of Medical Microbiology and Parasitology, University College Hospital; 16 Department of Community Medicine, College of Medicine, University of Ibadan; 17 Department of Community Medicine, University College Hospital, Ibadan, Nigeria; 18 Department of Microbiology and Immunology, KU Leuven; 19 Department of Clinical Sciences, Institute of Tropical Medicine, Antwerp, Belgium; 20 Institut National de Recherche Biomedicales, Kinshasa; 21 Service de Microbiologie, Cliniques Universitaires de Kinshasa, Democratic Republic of Congo; 22 Institute of Tropical Medicine, Eberhard-Karls University of Tübingen, Germany; 23 Duy Tan University, Da Nang, Vietnam; 24 Kilimanjaro Christian Medical Centre, Moshi, Tanzania; 25 Division of Infectious Diseases and International Health, Duke University Medical Center; 26 Duke Global Health Institute, Duke University, Durham, North Carolina; 27 Centre for International Health, University of Otago, Dunedin, New Zealand; 28 Jenner Institute, University of Oxford, United Kingdom; 29 Clinical Sciences, Liverpool School of Tropical Medicine; 30 Centre for Tropical Medicine, Nuffield Department of Medicine, University of Oxford, United Kingdom; 31 National Center for Emerging and Zoonotic Infectious Diseases, Centers for Disease Control and Prevention, Atlanta, Georgia; 32 icddr,b, Dhaka, Bangladesh; 33 Fielding School of Public Health, University of California, Los Angeles

**Keywords:** Severe typhoid fever, invasive Salmonellosis, host immunity and carriage, surveillance protocol, sub-Saharan Africa

## Abstract

**Background:**

Invasive salmonellosis is a common community-acquired bacteremia in persons residing in sub-Saharan Africa. However, there is a paucity of data on severe typhoid fever and its associated acute and chronic host immune response and carriage. The Severe Typhoid Fever in Africa (SETA) program, a multicountry surveillance study, aimed to address these research gaps and contribute to the control and prevention of invasive salmonellosis.

**Methods:**

A prospective healthcare facility–based surveillance with active screening of enteric fever and clinically suspected severe typhoid fever with complications was performed using a standardized protocol across the study sites in Burkina Faso, the Democratic Republic of Congo (DRC), Ethiopia, Ghana, Madagascar, and Nigeria. Defined inclusion criteria were used for screening of eligible patients for enrollment into the study. Enrolled patients with confirmed invasive salmonellosis by blood culture or patients with clinically suspected severe typhoid fever with perforation were eligible for clinical follow-up. Asymptomatic neighborhood controls and immediate household contacts of each case were enrolled as a comparison group to assess the level of *Salmonella*-specific antibodies and shedding patterns. Healthcare utilization surveys were performed to permit adjustment of incidence estimations. Postmortem questionnaires were conducted in medically underserved areas to assess death attributed to invasive *Salmonella* infections in selected sites.

**Results:**

Research data generated through SETA aimed to address scientific knowledge gaps concerning the severe typhoid fever and mortality, long-term host immune responses, and bacterial shedding and carriage associated with natural infection by invasive salmonellae.

**Conclusions:**

SETA supports public health policy on typhoid immunization strategy in Africa.

Invasive salmonellosis in humans is largely caused by fecal-oral transmission of *Salmonella enterica* subspecies *enterica* serovars Typhi (*S.* Typhi) and Paratyphi A (*S.* Paratyphi A), typically resulting in systemic typhoid fever (TF) and paratyphoid fever (PF), and nontyphoidal *S. enterica* (NTS) serovars causing self-limiting enterocolitis and bacteremia among children and adults in sub-Saharan Africa. Globally, TF accounts for 21.7 million cases and 217 000 deaths annually whereas invasive nontyphoidal *Salmonella* (iNTS) disease accounts for 3.4 million cases and >680 000 deaths [1, 2]. More recent systematic reviews of the burden of TF in low- and middle-income countries (LMICs) suggest 20.6 million cases and 223 000 deaths [3, 4], and adjusted for water-related risks and diagnostic factors, 11.9 million cases and 129 000 deaths [3]. A meta-regression analysis further estimated 17.8 million TF cases to occur each year in LMICs [5]. A recent multicountry TF surveillance study in Africa identified children <15 years and 3 years old as the prime risk groups for TF and iNTS disease, respectively [6]. Antimicrobial-resistant (AMR) and multidrug-resistant (MDR) TF and iNTS disease are increasingly reported from this region, highlighting the need for safe and effective vaccines and immunization strategies, particularly in countries with high prevalence of AMR/MDR typhoid and iNTS disease [7–10].

Currently available typhoid vaccines include the parenteral unconjugated Vi polysaccharide (ViPS) and oral live attenuated Ty21a vaccines, both of which have been recommended by the World Health Organization (WHO) since 2008, and parenteral typhoid conjugate vaccine (TCV), which was prequalified by the WHO in December 2017 [11–13]. Infants <2 years old and children <6 years old for whom ViPS and Ty21a vaccines, respectively, were not licensed, can now be immunized with TCV, which is licensed and recommended for infants aged 6 months or older [11, 14, 15]. Booster vaccinations are recommended for recipients of ViPS (every 2–3 years) and Ty21a (every 3–7 years) in typhoid-endemic settings, but further studies are needed to inform the need for TCV boosting [11, 16]. No iNTS or paratyphoid vaccine is currently available. Because children and infants are at high risk from typhoid and iNTS disease in many sub-Saharan African countries, advancement of these vaccines is warranted in support of Sustainable Development Goal 3 [17], as well as a better understanding of disease burden and severity. Several publications suggest that AMR/MDR and clinical factors such as hypothermia and anemia are associated with TF mortality [18, 19].

However, there is a paucity of population-based data concerning the incidence and severity of typhoid and iNTS disease among children and adults in sub-Saharan Africa. The Severe Typhoid Fever in Africa (SETA) program primarily aimed to understand the burden of severe TF and the associated case fatalities, clinical characteristics, and potential host risk factors that may be related to the disease severity. The SETA program also aimed to investigate the host immune response and bacterial shedding patterns associated with invasive salmonellosis. Public and private cost burden and productivity loss due to the treatment of respective diseases were further studied. Generated data will be essential in developing adequate immunization strategies and typhoid and iNTS disease control and prevention policies. These SETA study results will have a direct impact, particularly in countries eligible for support from Gavi, the Vaccine Alliance, on potential uptake of TCV in the next 10 years [20].

## METHODS

### SETA Study Objectives

The SETA program investigated (1) the burden and severity of invasive *Salmonella* infections (prospective surveillance with active screening at selected healthcare facilities); (2) host immunity and acute and chronic carriage associated with natural *S.* Typhi/*S.* Paratyphi A, B, and C (hereafter *S.* Paratyphi)/iNTS infections over a 1-year follow-up period (prospective case-controlled and cohort study design method); (3) prevalences of *S.* Typhi/*S.* Paratyphi/NTS carriage in immediate household members of confirmed TF, PF, and iNTS disease cases (prospective active surveillance and cohort study); (4) public and private expenditures for treatment and productivity loss (cost of illness) associated with TF/PF/iNTS disease (cohort) (5) effects of invasive salmonellosis on the quality of life of patients and long-term socioeconomic study (cohort); and (6) validation of a new reverse-transcription polymerase chain reaction (rt-PCR) assay for the diagnosis of invasive *Salmonella* infections (Table 1). In addition to TF/PF/iNTS disease, other etiologies of bacteremia, *Plasmodium* infections, and viral hepatitides were also sought, where feasible, through testing at the study healthcare facilities and laboratories.

**Table 1.** Objectives and Outcomes of the Severe Typhoid Fever in Africa Program

Objective	Outcome
1. To estimate the burden and severity of invasive *Salmonella* infections	a. Population-based adjusted incidence of invasive *Salmonella* infections b. Incidence of hospital- and community-based complications c. Mortality rate and long-term sequelae of *Salmonella* Typhi infections over 1 year of follow-up d. Prevalence of antimicrobial resistance among *Salmonella* isolates identified
2. To assess the immune response and carriage associated with natural TF, PF, and iNTS infection over a 1-year follow-up period	a. Assessment of the magnitude and duration of the immune response to TF, PF, and iNTS after natural infection b. Identification and validation of immunological markers associated with the development of *Salmonella* Typhi, *Salmonella* Paratyphi, and iNTS carriage
3. To estimate the prevalence of *S.* Typhi, *S.* Paratyphi, and NTS carriers among immediate household members of positive *Salmonella* cases	a. Prevalence of *S.* Typhi, *S.* Paratyphi, and NTS carriers among immediate household members of positive cases b. Immunological characterization of *S.* Typhi, *S.* Paratyphi, and NTS carriers c. Description of circulating *S.* Typhi, *S.* Paratyphi, and NTS isolates in the community and blood culture–confirmed cases
4. To estimate public and private expenditures for treatment and productivity loss associated with illness due to TF, PF, and iNTS infections^a^	a. Calculation of per-case direct and indirect cost of illness for TF, PF, and iNTS, categorized by payer and stratified by age and type of service used
5. To estimate the effects of invasive salmonellosis on the quality of life of patients and the subsequent disease-related family and societal burdens over a 1-year follow-up period^a^	a. Assessment of the quality of life of patients affected with invasive salmonellosis compared to a control group over a 1-year follow-up period
6. To validate a new rt-PCR assay for the diagnosis of invasive *Salmonella* infections in selected SETA sites^b^	a. Validation of rt-PCR assay for the diagnosis of invasive *Salmonella* infections

Abbreviations: iNTS, invasive nontyphoidal *Salmonella*; NTS, nontyphoidal *Salmonella*; PF, paratyphoid fever; rt-PCR, reverse-transcription polymerase chain reaction; SETA, Severe Typhoid Fever Surveillance in Africa program; TF, typhoid fever.

^a^Not applicable for Nigeria and the Democratic Republic of Congo.

^b^Only for Burkina Faso and Ghana.

### Study Sites

**Figure 1.**
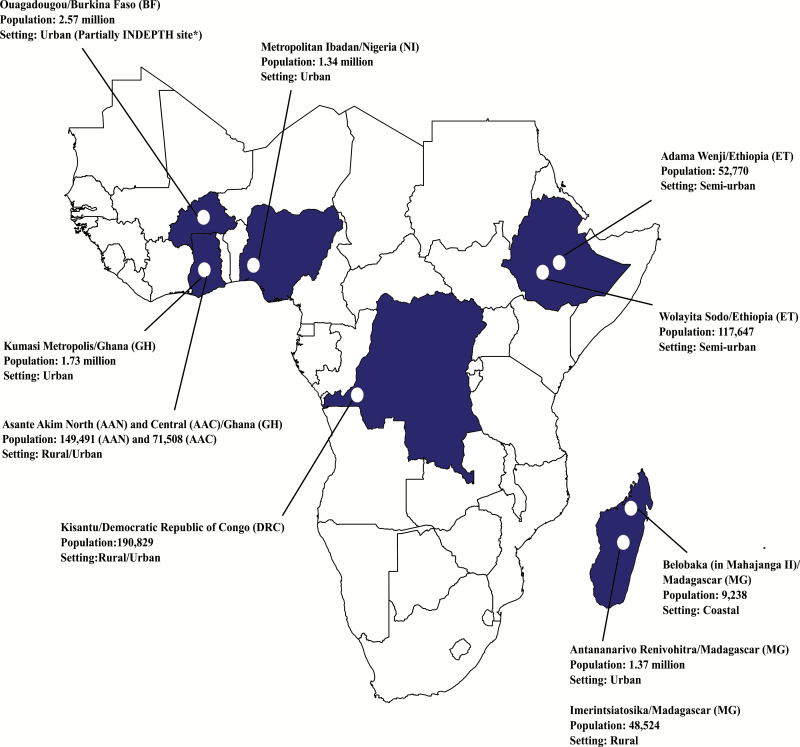
Locations of the Severe Typhoid Fever in Africa program sites. Population data sources: Burkina Faso: United Nations population division, Department of Economic and Social Affairs, 2014; Ouagadougou Health and Demographic Surveillance System routine data, 2015 (NiokoII/Polesgo nested in Ouagadougou); International Network for the Demographic Evaluation of Populations and Their Health. Democratic Republic of Congo: Kisantu Central Health Zone Office, 2017 (Nkandu/Kavwaya nested in Kisantu). Ethiopia: 2016 Ethiopia Health Management Information System, Ministry of Health. Ghana: Agogo Presbyterian Hospital official catchment area (Asante Akim North); 2010 Population and Housing Census (Asante Akim Central and Kumasi Metropolis). Madagascar: Ministry of Health, Repartition de la population par Fokotany, 2018 (Imerintsiatosika); Madagascar Population Statistics from Institut National de la Statistique de Madagascar (National Institute of Statistics)/United Nations Population Fund, 2015 (Antananarivo); commune census, 2019 (Belobaka). Nigeria: Annual Abstract of Statistics 2011, National Bureau of Statistics, Federal Republic of Nigeria. Abbreviation: INDEPTH, International Network for the Demographic Evaluation of Populations and Their Health.

Building on the Typhoid Fever Surveillance in Africa Program (TSAP) network [6], the SETA program utilized and expanded on previously established fever surveillance infrastructure in sub-Saharan Africa. Six countries were selected exhibiting high disease endemicity (Burkina Faso, Ghana, Madagascar), further need for in-country investigations on TF (Ethiopia), and value of extending to additional study sites to countries with large population numbers (Democratic Republic of Congo and Nigeria); study sites in these countries have been integrated into the SETA program to enable harmonized multicountry surveillance and data comparability (Figure 1). Each SETA site has two distinct study areas (Table 2): a medically served area where surveillance and subsequent studies (case follow-up, enrollment and follow-up of neighborhood controls [NCs] and household contacts [HCs], healthcare utilization survey, and cost of illness and long-term socioeconomic studies) were performed, and a medically underserved area where the frequency of mortality due to suspected severe TF was assessed through postmortem questionnaires. The overall study period was between 2016 and 2019, but varies per site (Table 2).

**Table 2.** Selected Sites, Healthcare Facilities, and Collaborating Institutions of the Severe Typhoid Fever in Africa Program

Country^a^	Study Site	Setting	Catchment population size^b^ No. (Year)	Healthcare facility	Healthcare facility type	Study period^c^	Collaborating Institution	Site Laboratory
Burkina Faso	Ouagadougou	Urban	2.57 million (2014)	a. Yalgado Hospital	Tertiary	Sep 2016-Jun 2019	ISSP	Schiphra Hospital Laboratory
				b. Charles de Gaulle Hospital	Pediatric Tertiary	Dec 2016-Jun 2019		
				c. Kossodo Hospital	Secondary	May 2016-Dec 2020		
				d. Polesgo Health Care Center	Primary	May 2016-Dec 2020		
	Balé	Rural	216,194 (2006)	Medically under-served area	...	Apr 2017-Oct 2018		
Democratic Republic of Congo (DRC)^**d**^	Kisantu	Rural & Urban	190,829 (2017)	a. Kisantu Hospital Saint-Luc	Tertiary	Sep 2017-Jan 2020	NIBR	Saint Luc Kisantu Hospital Laboratory
				b. Nkandu 1 Health Center	Primary	Jan 2018-Jan 2020		
				c. Kavuaya Health Center	Primary	Jan 2018-Jan 2020		
	n/a	n/a	n/a	Medically under-served area: n/a	n/a	n/a		
Ethiopia	Wolayita Sodo	Semi-urban	117,647 (2016)	a. Sodo Health Center	Primary	Jul 2017-Sep 2019	AHRI	Sodo Teaching Hospital; Adama Regional Laboratory
				b. Sodo Teaching Hospital	Secondary/ Tertiary	Jul 2017-Sep 2019		
				c. Sodo Christian Hospital	Secondary/ Tertiary	Jul 2017-Sep 2019		
	Adama Wenji	Semi-urban	52,770 (2016)	a. Shewa Alem Tena Health Center	Primary	Aug 2017-Sep 2019		
				b. Gefersa Health Center	Primary	Aug 2017-Sep 2019		
				c. Kuriftu Health Center	Primary	Aug 2017-Sep 2019		
				d. Adama Hospital	Secondary/ Tertiary	Aug 2017-Sep 2019		
	n/a	n/a	n/a	Medically under-served area: n/a	n/a	n/a		
Ghana	AAN & AAC	Rural & Urban	220,999 (2010)	a. Agogo Presbyterian Hospital	Secondary	May 2016-May 2019	KCCR; KNUST	KCCR; KNUST
	Kumasi Metropolis	Urban	1.73 million (2010)	a. Komfo Anokye Teaching Hospital	Tertiary	May 2016-May 2019		
	Tontokrom Keniago	Rural Rural	19,382 (2010) 15,808 (2010)	Medically under-served area Medically under-served area	... ...	July 2016-Dec 2017 July 2016-Dec 2017		
Madagascar	Antananarivo Renivohitra	Urban	1.37 million (2015)	a. Centre Hospitalier Universitaire d’Antananarivo- Hopital Joseph Ravoahangy Andrianavalona (HJRA)	Tertiary	May 2016-July 2019	UOA	UOA
				b. Centre Hospitalier Universitaire Joseph Raseta Befelatanana	Secondary/ Tertiary	June 2016-July 2019		
				c. Centre Hospitalier Universitaire Mere Enfant Tsaralalana	Pediatric Secondary/ Tertiary	June 2016-July 2019		
	Imerintsiatosika	Rural	48,524 (2018)	a. Imerintsiatosika Centre de Santé de Base II (CSBII)	Primary	Feb 2016-July 2019		
	Belobaka (in Mahajanga II)	Coastal	9,238 (2019)	a. Belobaka Centre de Santé de Base II (CSBII)	Primary	June 2018-July 2019		
	Andina commune Ilaka commune Tsarasaotra commune Antoetra commune	Rural Rural Rural Rural	24,425 (2015) 18,230 (2015) 23,290 (2015) 15,051 (2014)	Medically under-served area Medically under-served area Medically under-served area Medically under-served area	... ... ... ...	Mar 2016-July 2019 Mar 2016-July 2019 Mar 2016-July 2019 Mar 2016-July 2019		
Nigeria	Metropolitan Ibadan	Urban	1.34 million (2011)	a. University College Hospital	Tertiary	Feb 2017-July 2019	UOI College of Medicine; University College Hospital	University College Hospital, Department of Medical Microbiology and Parasitology
				b. Our Lady of Apostles Catholic Hospital Oluyoro	Secondary	April 2017-July 2019		
				c. Adeoyo Maternity Teaching Hospital	Secondary	May 2017-July 2019		
				d. Kola Daisi Foundation Community Health Centre	Primary	April 2017-July 2019		
	Ibarapa North	Semi-urban	121,860 (2011)	Medically under-served area	...	May 2018-July 2019		

Abbreviations: DRC, Democratic Republic of Congo; n/a, not available; AAN, Asante Akim North; AAC, Asante Akim Central; ISSP, Institut Superieur des Sciences de la Population; NIBR, National Institute of Biomedical Research; AHRI, Armauer Hansen Research Institute; KCCR, Kumasi Center for Collaborative Research; KNUST, Kwame Nkrumah University of Science and Technology; UOA, University of Antananarivo; UOI, University of Ibadan

^a^Ethical approval: International Vaccine Institute Institutional Review Board (IRB No. 2015-006); Institute of Tropical Medicine Antwerp Institutional Review Board, Belgium; Universiteit Antwerpen, Comite voor Medische Ethiek, Belgium; Ministère de la Santé du Burkina Faso, Comité d’Ethique pour la Recherche en Santé, Burkina Faso; Comité d’Ethique de l’Ecole de Santé Publique de l’Université de Kinshasa, Democratic Republic of Congo (No ESP/CE/011/2017); National Research Ethics Review Committee (NRERC), Ministry of Science and Technology, Federal Democratic Republic of Ethiopia; AHRI/All African Leprosy, Tuberculosis and Rehabilitation Training Center (ALERT) Ethics Review Committee (AAERC), Ethiopia; National Research Ethics Review Committee (NRERC), Ethiopia; Kwame Nkrumah University of Science and Technology, School of Medical Sciences/Komfo Anokye Teaching Hospital, Committee on Human Research, Publication and Ethics, Ghana; Ministère de la Santé du Repoblikan’l Madagaskar, Comité d’Ethique, Madagascar;University of Ibadan/University College Hospital Ethics Committee (No. UI/EC/16/0369), Ibadan, Nigeria; Ethics Committee, Our Lady of Apostles Catholic Hospital Oluyoro (OLA) (No. OCH/EC/17/05), Ibadan, Nigeria; Oyo State Ethics Review Committee (AD13/479/665A), Nigeria.

^b^Source of population data: see Figure 1. Population data of the medically under-served areas in Madagascar are from the respective commune census.

^c^Study period for the surveillance activities including the enrolment of eligible participants and follow-ups.

^d^The study duration in the DRC and Nigeria may be extended until July 2020 for additional one year surveillance activities.

Surveillance sentinel sites were established including primary, secondary, and tertiary healthcare facilities (Table 2). Selection of healthcare facilities was based on previous documentation of the occurrence of TF/PF/iNTS disease; case reports or series of patients with severe TF; capacity to conduct blood culture and epidemiological research; accessibility of local population to the healthcare facilities; and preference of healthcare facilities by the local population in the event of febrile illness. Each study site was characterized by having access to at least one tertiary hospital and at least one secondary or primary healthcare facility where patients were enrolled into the SETA program. Surveillance catchment areas were predefined based on the geographic or administrative coverage of healthcare services provided by respective healthcare facilities (hospital records or information materials). Healthcare-seeking behavior data collected during TSAP and other existing national census and/or demographic data, generated through the Health and Demographic Surveillance System (HDSS) under the International Network for the Demographic Evaluation of Populations and Their Health (INDEPTH), were also considered [21, 22].

### Prospective Sentinel-based Surveillance With Active Screening

Patients visiting SETA sentinel healthcare facilities were actively screened at selected entry points of each healthcare facility, including triages where applicable, outpatient wards, inpatient medical and surgical wards, and emergency wards. Laboratories of the selected healthcare facilities/collaborating institutes were involved for laboratory-based detection of any invasive *Salmonella* from clinical samples such as blood and specimens from perforation cases, which may be collected outside of the routine SETA patient screening procedure. The generic SETA study forms are available in the Supplementary Data.

### Inclusion Criteria and Specimen Collection

Patient screening for eligibility was based on the following inclusion criteria (Table 3): history of fever (≥3 consecutive days in the last seven days prior to visiting the healthcare facility), or current objectively assessed fever (≥38°C tympanic/rectal and/or ≥37.5°C axillary), or clinically suspected TF or blood culture positive for invasive salmonellosis, or gastrointestinal perforations. Residency within the predefined catchment area was also an inclusion criterion, except for patients with clinically suspected severe TF complications such as gastrointestinal perforations who were eligible for enrollment regardless of catchment residence (special cases; Table 4). Blood and stool samples were collected for culture from eligible patients at enrollment to investigate causative pathogens, antimicrobial resistance, and acute carriage (shedding), and urine samples were examined for antimicrobial pretreatment status (Table 5). Oropharyngeal swab samples were collected additionally for a supplemental investigation of group A *Streptococcus* carriage.

**Table 3.** Inclusion Criteria

Patient enrollment in tertiary healthcare facilities: 1. Fever reported for ≥3 consecutive days within the last 7 days in patients living in the defined catchment area, *OR* 2. Patients with clinically suspected TF living in the defined catchment area, *OR* 3. Blood culture positive for *Salmonella* Typhi/*Salmonella* Paratyphi/iNTS (outside SETA) in patients living in the defined catchment area, *OR* 4. Pathognomonic gastrointestinal perforations (ie, clinically diagnosed TF gastrointestinal perforation), even in the absence of laboratory confirmation, in patients living in and outside the defined catchment area (special cases), *AND* 5. Informed consent form signed
Patient enrollment in primary and secondary healthcare facilities: 1. Patients living in the defined catchment area presenting to healthcare facility with objective fever of ≥38°C tympanic/rectal AND/OR ≥37.5°C axillary, *OR* 2. Fever reported for ≥3 consecutive days within the last 7 days in patients living in the defined catchment area, *OR* 3. Patients with clinically suspected TF living in the defined catchment area, *OR* 4. Blood culture positive for *S.* Typhi/*S.* Paratyphi/iNTS (outside SETA) in patients living in the defined catchment area, *OR* 5. Pathognomonic gastrointestinal perforations (ie, clinically diagnosed TF gastrointestinal perforation), even in the absence of laboratory confirmation, in patients living in and outside the defined catchment area (special cases), *AND* 6. Informed consent form signed
Neighborhood controls: 1. Age (±5 years), sex, and residency (neighborhood) matched to *S.* Typhi/*S.* Paratyphi/iNTS disease and special cases, *AND* 2. No subjective or objective fever at any point within 28 days prior to enrollment, *AND* 3. No subjective or objective fever on the date of case enrollment (“focal time”), *AND* 4. Informed consent form signed
Household contacts: 1. Immediate household contacts of *S.* Typhi/*S.* Paratyphi/iNTS disease and special cases, *AND* priority should be given to: (1) Individual(s) who prepares food for the case (2) Individual(s) closest in age to the case (3) Individual(s) who spends the most time with the case Alternative household contacts should be enrolled if individuals meeting the above priority do not wish to participate or are not identified. 2. Informed consent form signed

Abbreviations: iNTS, invasive nontyphoidal *Salmonella*; SETA, Severe Typhoid in Africa program; TF, typhoid fever.

**Table 4.** Case Definitions

Case Type	Definition
Confirmed TF case	Positive blood culture for *Salmonella* Typhi
Mild TF case	Blood culture–confirmed TF without any complication of TF listed in Table 6
Severe TF case^a^	Blood culture confirmed TF with any one complication(s) of TF listed in Table 6
Special case^b^	Pathognomonic gastrointestinal perforations (ie, clinically diagnosed TF gastrointestinal perforation), even in the absence of laboratory confirmation, in patients living in and outside the defined catchment area
Confirmed PF case	Patients with a positive blood culture for *Salmonella* Paratyphi serovars
Confirmed iNTS disease case	Patients with a positive blood culture for any nontyphoidal *Salmonella* serovar
Relapse	Blood culture–confirmed case who becomes ill with a subsequent episode of TF/PF/iNTS disease within 90 days of the prime infection. (A relapse should be a discernably similar or identical infecting strain, for which further genomic analyses will be performed.)
Reinfection	Blood culture–confirmed case who becomes ill with a subsequent episode of TF/PF/iNTS disease on or after a 90-day period after documentation of a previous blood culture–confirmed TF/PF/iNTS disease. (A reinfection should be a discernably different infecting strain, for which further genomic analyses will be performed.)
*S.* Typhi/*S.* Paratyphi/NTS carrier	An individual shedding *S.* Typhi or *S.* Paratyphi or NTS in stool after symptom resolution.

Abbreviations: iNTS, invasive nontyphoidal *Salmonella*; NTS, nontyphoidal *Salmonella*; PF, paratyphoid fever; TF, typhoid fever.

^a^Severe typhoid: Refer to Table 6 for complications associated with TF.

^b^Special case: Refer to Table 3 for study inclusion criteria.

**Table 5.** Follow-Up Schedule and Sample and Data Collection for Seta Participants

Enrolment of eligible patients	Follow-up of Study participants	Follow-up Schedule^b^						
Day 0^a^		Day 3–7^c^	Day 12–14	Day 28–30	Day 90	Day 180	Day 270	Day 365
Blood Stool OPS^d^ Urine LT-SES (QoL only)^e^	*Salmonella* cases/ special cases	Blood Stool OPS (only iNTS case) LT-SES COI^f^ COOI^f^	... ... ... LT-SES COI COOI	Blood Stool OPS (only iNTS case) LT-SES COI COOI	Blood Stool OPS (only iNTS case) LT-SES COI COOI	Blood Stool OPS (only iNTS case) LT-SES ... ...	... ... ... LT-SES .. ...	Blood Stool OPS (only iNTS case) LT-SES ... ...
	Neighborhood controls (NCs)^**g**^	Enrolment				Follow-up^**h**^		
		Blood Stool OPS (only iNTS associated)	... ... ...	... ... ...	... ... ...	Blood Stool OPS (only iNTS associated)	... ... ...	Blood Stool OPS (only iNTS associated)
		LT-SES COOI^f^	LT-SES COOI^f^	LT-SES COOI^f^	LT-SES COOI^f^	LT-SES ...	... ...	LT-SES ...
	Household contacts (HCs)^g^	Enrolment				Follow-up^**h**^		
		Blood Stool OPS (only iNTS associated)	... ... ...	... ... ...	... ... ...	Blood Stool OPS (only iNTS associated)	... ... ...	Blood Stool OPS (only iNTS associated)
	Clinical cases (CCs)^i^	COI^f^	COI^f^	COI^f^	COI^f^	...	...	...

Abbreviations: COI, cost of illness; COOI, Cost of other illness; LT-SES, long-term socioeconomic study; iNTS, invasive nontyphoidal *Salmonella*; OPS, oropharyngeal swab; QoL, Quality-of-Life.

^a^Day 0: Enrolment date of the SETA study-eligible patients who meet the study inclusion criteria (See Table 3).

^b^Clinical follow-up visits for cases: visit 1 (Day 3–7 or as soon as blood culture confirmation), visit 2 (days 28–30), visit 3 (day 90), visit 4 (day 180), and visit 5 (day 365) with a window period of *+*7 days (or longer as appropriate to address challenges in the respective study field settings).

^c^Day 3–7: Date when blood culture result is known. This time point may not be strictly limited as scheduled.

^d^OPS collection at enrolment and for only iNTS cases and the corresponding matched neighborhood controls (NCs) and household contacts (HCs). Country-specific adjustments may be further applied.

^e^LT-SES and COI surveys are performed in parallel to the clinical follow-up visits of cases and NCs, with additional follow-up time points (days 12–14 and day 270). LT-SES: Quality-of-Life (QoL) and Long-term Socio-Economic Study surveys.

^f^COI & COOI stops when self-reported illness ends. Only *S*. typhi cases, special cases and NCs receive COOI.

^g^Enrolment of NCs and HCs are recommended during the first follow-up visit of the corresponding cases, which is day 3–7 or as soon as blood culture confirmation of cases.

^h^Clinical follow-up visits for NCs and HCs after enrolment: visit 1 (day 180) and visit 2 (day 365).

^i^Clinical cases (CCs): Clinical cases are laboratory negative but clinical suspected typhoid fever cases. CCs are matched with *S*. Typhi cases and special cases.

### Severe Typhoid

Daily progress of clinical symptoms and treatment history were recorded for all enrolled patients with clinically suspected severe TF (Table 4) until discharge, regardless of blood culture results. Patients with severe TF complications (Table 6) requiring and/or undergoing surgery were closely monitored by hospital physicians, who gauged the willingness of the patients to participate in this research on severe typhoid by providing sufficient information and accurate explanation concerning the study activities and expectations. Patient enrollment only occurred after written informed consent was obtained. In case of emergencies whereby patients with gastrointestinal perforations required an immediate surgical intervention, an existing hospital consent process/form for surgery was followed for patient treatment without delay. Such patients were approached after stabilization for informed consent for study participation. Where feasible and based on patient or/and guardian consent, treating physicians collected blood and/or surgical samples such as gastrointestinal tissue, gallbladder, bile, or peritoneal fluid for culture and/or PCR. Histopathology and cytology of surgical samples were performed at the hospital, if feasible.

**Table 6.** Possible Systemic Complications of Typhoid Fever

Complication	Definition		
Gastrointestinal bleeding^a^	The presence of visible blood or melena in the stool with a positive fecal occult blood test		
Gastrointestinal perforation	Gastrointestinal perforation in the vicinity of the terminal ileum (or ileum/cecum/colon) typical of typhoid and seen at laparotomy (if available)		
Encephalopathy^b^	Patients with any of the following aspects of altered mental status: (1) Delirium: markedly confused thinking and speech; (2) Obtundation: patient who appears unconscious but can be stimulated to respond appropriately to questions and comments; (3) Stuporose: patient who does not respond appropriately to any stimuli but withdraws appropriately to noxious stimuli; (4) Comatose: patient who does not respond appropriately to noxious stimuli. These exclude patients with disorientation and poor short-term memory but not delirium; apathetic or lethargic without obtundation *OR* GCS score ≤12 and/or Blantyre score <5 without alternative diagnosis *AND* with CSF examination within normal limits (no WBC and a normal CSF glucose and protein)		
Meningitis	Symptoms suggestive of meningitis and an abnormal CSF examination with/without *Salmonella* Typhi or iNTS or *Salmonella* Paratyphi A isolated from CSF culture		
Hemodynamic shock^a^	Systolic blood pressure <90 mm Hg in patients aged ≥12 y or <80 mm Hg in patients aged <12 y with clinical evidence of tissue hypoperfusion (ie, abnormal state of consciousness; cold and clammy skin; constricted peripheral veins; oliguria [<20 mL urine/h] after rehydration)		
Myocarditis^a^	Abnormal cardiac rhythm or abnormal ECG as interpreted by physician; ultrasound evidence of a pericardial effusion; ventricular failure		
Hepatitis^a^	Visible jaundice and/or hepatomegaly with abnormal levels of serum SGOT (AST) (>400 IU/L) and/or SGPT (ALT) (>400 IU/L) or >5 times the ULN of liver enzyme tests		
Cholecystitis^a^	Right upper quadrant pain and tenderness without evidence of hepatitis; ultrasound evidence of enlarged gall bladder or gall bladder with thickened wall		
Pneumonia^a^	Respiratory symptoms (eg, cough) with abnormal chest radiograph infiltrates		
Pleural effusion	Clinical (ie, shortness of breath, chest pain) and radiological evidence of a pleural effusion		
Anemia^c^	Moderate: Hb 7.0–9.9 g/dL in children aged 6–59 mo Hb 8.0–10.9 g/dL in older children and adults	Severe: Hb <7.0 g/dL in children aged 6–59 mo Hb <8.0 g/dL in older children and adults	
Focal infection	Abscess or collection at a specific site (eg, spleen, joint, bone) with *Salmonella* Typhi or *Salmonella* Paratyphi A isolates from drainage culture		
Renal impairment	Creatinine >2 mg/dL *OR* 175 μmol/L		

Abbreviations: ALT, alanine aminotransferase; AST, aspartate aminotransferase; CSF, cerebrospinal fluid; ECG, electrocardiogram; GCS, Glasgow Coma Scale; Hb, hemoglobin; SGOT, Serum Glutamic Oxaloacetic Transaminase; SGPT, Serum Glutamic Pyruvic Transaminase; ULN, upper limit of normal; WBC, white blood cell.

^a^Ndila C, Bauni E, Mochamah G, et al. Causes of death among persons of all ages within the Kilifi Health and Demographic Surveillance System, Kenya, determined from verbal autopsies interpreted using the InterVA-4 model. Glob Health Action 2014; 7:25593.

^b^Leung DT, Bogetz J, Itoh M, et al. Factors associated with encephalopathy in patients with *Salmonella enterica* serotype Typhi bacteremia presenting to a diarrheal hospital in Dhaka, Bangladesh. Am J Trop Med Hyg 2012; 86:698–702.

^c^World Health Organization; Chan M. Haemoglobin concentrations for the diagnosis of anaemia and assessment of severity. Geneva, Switzerland: WHO, 2011:1–6.

Assessment of death attributed to invasive *Salmonella* infection, particularly TF, was also analyzed through postmortem questionnaires (see Supplementary Data) performed in the medically underserved areas in selected study sites. While the *International Statistical Classification of Diseases and Related Health Problems*, *Tenth Revision* (*ICD-10*) [23] and the verbal autopsy standards manual [24] recommended by the WHO were referenced, the SETA postmortem questionnaires adapted symptoms outlined in the case definition, which included prolonged history of fever before deterioration and death, at least 1 abdominal-related symptom, deterioration consistent with the known TF complications such as acute abdomen or gastrointestinal bleeding or encephalopathic development with gradual deterioration to death, and history of treatment or hospitalization. This questionnaire was also administered to participants enrolled in the medically served areas in case of any fatality occurring during the study period.

### Host Immunity and Carriage Associated With Invasive Salmonellosis (Cases)

Enrolled patients with blood culture–confirmed TF/PF/iNTS (cases) became part of the study cohort for clinical follow-up and health economics surveys during the period of 1 year (Table 5). At each clinical follow-up visit, blood and stool samples were collected from all cases and additional oropharyngeal swab samples were obtained from iNTS cases. Immunological, biochemical, and parasitological assessments were conducted on the collected blood samples (immunoglobulin G [IgG]/immunoglobulin M [IgM] antibodies, complete blood count, malaria, creatinine, bilirubin, alanine aminotransferase, aspartate aminotransferase, and additional peripheral blood mononuclear cell and T-cell stimulations where feasible in Ghana and Burkina Faso for a substudy on the cellular immune response associated with iNTS disease cases over the period of 1 year of follow-up). Stool culture was performed to detect bacterial shedding for assessment of acute and chronic carrier status up to the 1-year time point following the case confirmation [25, 26].

### Neighborhood Controls

For investigations of host immune response and carriage status of cases in the context of the local population, asymptomatic NCs matched to cases by age (±5 years), sex, and location of residency (1:4 case-control ratio) were enrolled (Table 3) following written informed consent. Neighborhood is defined based on the location of residency of TF/PF/iNTS cases within the surveillance catchment areas. Residency locations of cases noted for the study follow-up visits were applied to identify NCs, whereby neighbors residing within the same administrative units such as villages or communes were approached for age- and sex-matched criteria for screening and enrollment of NCs. Blood and stool samples were collected at enrollment and at days 180 and 360. Oropharyngeal swab samples were collected additionally from iNTS disease case-matched controls. Immunological assessments were conducted on the collected blood samples, and stools were cultured for *Salmonella*.

### Household Contacts

Upon identification of blood culture–positive TF/PF/iNTS cases, two immediate HCs were approached for study participation to investigate for carriage of *S.* Typhi, *S.* Paratyphi, and NTS. Household contacts are individuals residing in the same house as the case, sharing the common living condition and environment. Priority for enrollment was given to individuals who prepare food for the case, who is the closest in age to the case, and who spends the most time with the case (Table 3). Frequency and duration of *Salmonella* carriage was estimated through confirmation of bacterial shedding in stool over the period of 1 year. Blood samples were also collected from immediate HCs for the immunological characterization of *Salmonella* carriers (Table 4).

All samples collected and examined from study participants (cases/NCs/HCs) are described in Supplementary Table 1 (summary of sample collection for participants in SETA).

### Healthcare Utilization Survey

A healthcare utilization survey [27] was conducted in the surveillance catchment area to estimate the proportion of the population who seek healthcare at the SETA surveillance healthcare facilities in the event of any fever or symptoms consistent with severe enteric fever. This was used to derive age-specific adjustment factors to adjust crude incidence rates of TF, PF, and iNTS disease in the catchment population [21]. In addition, socioeconomic and water, sanitation, and hygiene (WASH)–related data collected through the survey allow further observational analyses on the surveillance catchment population and settings.

### Informed Consent and Ethical Considerations

All eligible participants (or their responsible parents/guardians) were approached for voluntary written informed consent. The informed consent process included an explanation by study staff about the study purpose, expectations from participants, duration of participation, risks and benefits for participation, confidentiality, right to decline or withdraw from the study, and contact information of study investigators. Participants were asked to sign a statement of consent if they agreed to join the study. If the participant was an infant or child, his or her parent/guardian was asked to sign or thumbprint the statement of consent. If the participant was an adolescent, both the participant and the parent/guardian were asked to sign or thumbprint the statement of consent. If the participant was illiterate, an independent literate witness (where possible, this person should be selected by the participant and had no connection to the study team) was asked to sign or thumbprint the statement of consent. Study participants were allowed to withdraw from the study at any time without loss of clinical services or penalties of any kind. The International Vaccine Institute’s (IVI) Institutional Review Board and site-specific ethical review boards reviewed the study annually to ensure continued compliance with the ethical principles and guidelines based on the WHO (2009) [28], the Council for International Organizations of Medical Sciences (2016) [29] and the Declaration of Helsinki (World Medical Association Declaration of Helsinki, 2013).

### Data Management

Data from each participant required complete and adequate source documentation (hospital or medical records, laboratory reports, test results) unless the data recorded directly on our study forms were considered the source data. The surveillance study forms included the informed consent and enrollment form for all participants, case report forms for patients, follow-up forms for *Salmonella-*confirmed patients or/and special cases and their NCs and HCs, postmortem questionnaires, and laboratory forms (Supplementary Data). Electronic data collection was performed using the SETA Collect software (Android 5.0.1; API 23) developed by the IVI for sites with stable internet access. In parallel, paper-based data collection was available at all sites. Data collected through SETA Collect were periodically exported to the IVI server via a web-based paperless data management system (PDMS). Paper-based data were entered into the computerized data management system (CDMS) using a dual entry process, which were transferred to the IVI monthly. This system automatically backed up data at systematic intervals onto local hard disks and external media and provided an audit trail. In the event of data discrepancy or missing values, study staff referred to the original source documents or contacted respective participants to clarify as needed. All tablets and PDMS/CDMS databases were password protected. All paper-based study forms were stored in cabinets with restricted access by authorized study staff. Both systems had algorithms for checking missing data points, range, and logical errors.

### Data Analysis Methodology and Plan

SETA provides incidence rates and frequency proportions of TF. The incidence estimation is based on the formula used in TSAP [6, 30], using adjustments such as the proportion of healthcare-seeking behavior in case of fever and severe febrile illnesses in the catchment population (denominator) and recruitment rate of study-eligible patients in the screened and enrolled patients (numerator) (Table 7). Equally, adjusted incidences, as well as frequency proportions of patients with severe TF (and deaths due to confirmed or/and suspected severe TF) out of the total number of TF cases are assessed. Final adjustment factors or multipliers to be applied in the adjusted incidence estimations are harmonized with the Severe Typhoid in Tanzania (STT) project [31] and the Surveillance for Enteric Fever in Asia Project (SEAP) [30, 32, 33] sites to ensure data comparability. A descriptive analysis of frequency and prevalence of deaths due to suspected severe TF from the selected medically underserved areas will be presented. The clinical history of symptoms associated with severe TF such as fever, abdominal pain, seizure, and other symptoms (Table 6) and the cause of death recorded, if known, are being made available. Due to the absence of autopsies, any analysis on death attributable to suspected severe TF complications will remain descriptive and limited.

**Table 7.** Formula for Incidence Estimations

Variable	Definition
n_i_	Number of cases *(S.* Typhi, iNTS, *S.* Paratyphi) during surveillance period in age group i
s_i_	Number of severe typhoid fever cases during surveillance period in age group i
1n_i_	Population present in catchment area at the start of surveillance in age group i
2n_i_	Estimated population present in catchment area at the end of surveillance in age group i
r_i_	Recruitment proportion of eligible patients in SETA healthcare facilities in age group i
h_i_	Proportion of population visiting SETA health care facilities (HCUS) in age group i
Adn_i_	Adjusted cases in age group i
Ads_i_	Adjusted cases of severe typhoid in age group i
PYO_i_	Population in person years observation in catchment area in age group i
APYO_i_ (Adjusted PYO)	Adjusted population at risk contributing to PYO in age group i (accounting for new, lost to follow-up, deceased individuals during surveillance period)
**Incidence of non-severe TF/PF/iNTS (TSAP-style** ^**a**^ incidence estimation using multipliers of healthcare utilisation and recruitment proportion)	Incidence per 100,000 = (new lab confirmed cases)/(population at risk) x 100,000 HDSS site: Numerator: Adn_i_ Adn_i_ = n_i_ x 1/r_i_ Denominator: APYO_i_ APYO_i_ = PYO_i_ x h_i_ Adjusted incidence rate_i_ in 100,000 PYO = (Adn_i_/APYO_i_) x 100,000 Non-HDSS site: Numerator: Adn_i_ Adn_i_ = n_i_ x 1/r_i_ Denominator: APYO_i_ 2n_i_ = 1n_i_ x (annual growth rate) x [(months of surveillance)/12] PYO_i_ = [(1n_i_+2n_i_)/2] x [(months of surveillance)/12] APYO_i_ = PYO_i_ x h_i_ Adjusted incidence rate_i_ in 100,000 PYO = (Adn_i_/APYO_i_) x 100,000
Incidence of severe TF	Number of severe typhoid fever cases during surveillance period in age group i: s_**i**_ Numerator: Ads_**i**_ Ads_i_ = s_i_ x 1/r_i_ Denominator: APYO_**i**_ APYO_i_ = PYO_i_ x h_i_ Incidence rate s_i_ in 100,000 = (severe TF cases)/(population at risk) x 100,000 Adjusted incidence rate s_i_ in 100,000 PYO = (Ads_i_/APYO_i_) x 100,000
Frequency proportion of severe TF	Number of severe typhoid fever cases out of total laboratory confirmed typhoid fever cases in percentages

Abbreviations: APYO, adjusted person-years of observation; HCUS, healthcare utilization survey; iNTS, invasive nontyphoidal *Salmonella*; NTS, nontyphoidal *Salmonella*; PF, paratyphoid

fever; PYO, person-years of observation; SETA, Severe Typhoid Fever in Africa program; TF, typhoid fever; TSAP, Typhoid Fever Surveillance in Africa Program.

^a^von Kalckreuth, V, Konings F, Aaby P, et al. The Typhoid Fever Surveillance in Africa Program (TSAP): Clinical, Diagnostic, and Epidemiological Methodologies. Clin. Infect. Dis 2016; 62:S9-S16.

Analyses on severe TF will also include investigations on host risk factors that may be associated with the severity of disease (eg, age group, sex, and comorbidities) and site-specific characteristics. Various statistical methodologies will be used including the Spearman correlation coefficient and/or stratified logistic regressions for univariate and multivariable analyses to measure the odds ratios. Data from the longitudinal cohort study of the TF/PF/iNTS patients and their respective NCs and HCs will be analyzed by comparing the level of antibodies (IgG/IgM) of cases and their shedding of *Salmonella* species during an acute phase and up to a 1-year time point, with that of NCs and HCs (background antibody level and bacterial shedding in the respective households and communities). *Salmonella* strains detected in stools of cases, HCs, and NCs will be analyzed to investigate any patterns potentially related to transmissions in respective study sites, for which the population-based healthcare utilization survey data will be cross-compared for various environmental risk factors such as the household WASH conditions and domestic animals. All bacterial isolates yielded through SETA will be sequenced for further molecular epidemiological analyses such as the emergence and spread of AMR/MDR strains as performed in TSAP [34]. Further analyses will be also performed including, but not limited to, the preuse of antimicrobials and/or antimalarials, AMR/MDR of detected bacterial pathogens, geospatial analyses of healthcare-seeking behavior and cases, socioeconomic and WASH risk factors, cost of illness, and long-term socioeconomic impact to patients.

## DISCUSSION

The SETA study protocol was aligned with the SEAP phase II [32, 33] and the STT project [31] under the recommendations of the Scientific Advisory Process for Optimal Research on Typhoid. Capacity building to support the existing public healthcare facilities selected as the SETA sentinel sites was essential for conducting a standardized surveillance in multiple sites in resource-limited settings. Active engagements of the healthcare professionals and field enumerators participating in the study were critical in ensuring a proper screening of eligible patients and reducing rates of missed screening or/and dropouts from the surveillance and follow-up activities. Applying various adjustment factors in a selective healthcare facility–based surveillance is considered to be a relatively low-cost hybrid surveillance method [30], whereby conducting a surveillance in all healthcare facilities in the study catchment area or/and an active community-based surveillance is not feasible.

Challenges were faced and addressed during the study period. These included the following: (1) introducing a complex research study with multiple study components, especially in tertiary hospitals, was challenging as the clinicians were occupied with their routine patient care; (2) contamination rates of blood culture varied per site; (3) volume of blood draw was challenging, particularly from children; (4) enrolling healthy controls in the neighborhood was also challenging and some loss to follow-up was inevitable over the course of 1 year; and (5) follow-up visit schedules were difficult to strictly adhere to, owing to various unforeseen and unexpected reasons such as the absence or refusal of the study participants. The SETA monitoring and evaluation plan and tool was developed and implemented throughout the study period (see Mogeni et al in this supplement) to ensure and improve the quality of surveillance across all sites.

The SETA program aimed to provide a comprehensive quantitative analysis of incidence, severity, complications, mortality, host immunity, and acute and long-term carriage, cost of illness, and socioeconomic burden of disease associated with invasive salmonellosis in multiple countries across the sub-Saharan African region. Upon completion of the study, data cleaning and analysis will be performed, and the results will be disseminated at academic conferences and international peer-reviewed journals. Observational descriptions of the surveillance study areas and populations including demographic and socioeconomic status, and WASH conditions and practices, will also provide a valuable dataset for the development of prevention policies for TF/PF/iNTS disease, including vaccination strategies, in the respective African countries. SETA results will further serve as baseline data for any future vaccination studies in these study sites.

## Supplementary Data

Supplementary materials are available at *Clinical Infectious Diseases* online. Consisting of data provided by the authors to benefit the reader, the posted materials are not copyedited and are the sole responsibility of the authors, so questions or comments should be addressed to the corresponding author.

ciz715_suppl_Supplementary_Table_1

## Notes

***Author contributions.*** J. D. C., S. E. P., and F. M. conceptualized the study design. R. F. B., E. D. M., C. M. P., C. A. M., J. A. C., Y. A. S., E. O. D., R. R., A. B. S., M. T., I. N. O., A. K., O. P., J. J., O. L. M., M. O., I. O., S. E., L. M. C. E., U. P., J. I., O. D. M., N. P., G. D. P., H. S., Y. C., V. M., E. R., A. D., and J. Y. P. further contributed in the study design. S. E. P. and T. T. drafted the protocol of the study and S. E. P. drafted this manuscript. All co-authors critically reviewed, revised, and approved the protocol and this manuscript.

***Financial support*.** This research was funded by the Bill & Melinda Gates Foundation [OPP1127988]. The International Vaccine Institute acknowledges its donors including the Republic of Korea and the Swedish International Development Cooperation Agency. This publication was made possible through a grant from the Bill & Melinda Gates Foundation [OPP1201031].

***Supplement sponsorship*.** This article was published as part of the supplement “Severe Typhoid Fever in Africa (SETA) Program” sponsored by the International Vaccine Institute.

***Potential conflicts of interest.*** The authors report no potential conflicts of interest. All authors have submitted the ICMJE Form for Disclosure of Potential Conflicts of Interest. Conflicts that the editors consider relevant to the content of the manuscript have been disclosed.
