# Supplementary material for: The Severe Typhoid Fever in Africa Program: Study Design and Methodology to Assess Disease Severity, Host Immunity, and Carriage Associated With Invasive Salmonellosis
Source: Clin Infect Dis. 2019 Oct 30;69(Suppl 6):S422–34. doi: 10.1093/cid/ciz715 (PMC6821161; doi:10.1093/cid/ciz715)
Supplement: ciz715_suppl_Supplementary_Table_1 [file ciz715_suppl_supplementary_table_1.docx]

**Supplementary Table1. Summary of sample collection for participants in the SETA program**

| **Sample** | **Recommended Sample amount** | **Test(s) performed** | **Test location** | **Sample processing** | **Storage at field depot^1^** | **Applicable participants^2^** | **Applicable countries^3^** |
| --- | --- | --- | --- | --- | --- | --- | --- |
| Blood | 1-5 mL^4^ | Blood culture | Field sites | Automated culture system | N/A^5^ | New born to ˂ 28kg b/w^6^ | All |
|  | 8-10 mL | Blood culture | Field sites | Automated culture system | N/A | ≥28kg b/w and adults | All |
|  | 1-3 mL | Blood counts/etc. | Field sites | N/A | N/A | Inclusion criteria | All |
|  | 1 drop | Malaria RDT^7^ | Field sites | N/A | N/A | Inclusion criteria | All (with site specific adjustments) |
|  | 1 drop | HIV^8^ testing | Field sites | N/A | N/A | TBD^9^ | TBD |
|  | 1 mL | Biobank/storage | IVI | Centrifuge | -80°C | Inclusion criteria | All |
|  | 1-3 mL | Rt-PCR^10^ assay | Field sites | TSB-Oxgall^11^ enrichment | N/A | Inclusion criteria | GH/BF |
|  | 8-10 mL | Immune assay | IVI/REF LAB^12^ | Centrifuge/PBMC^13^ separation | -80°C/LN2^14^ | ≥18 yr (TF/PF+) cases/NCs/HCs | All (with site specific adjustments) |
|  | 6-10 mL | Immune assay | IVI/REF LAB | Centrifuge/PBMC separation | -80°C/LN2 | ˂18 yr (TF/PF+) cases/NCs/HCs | All (with site specific adjustments) |
|  | 11-13 mL | Immune assay | IVI/REF LAB | Centrifuge/PBMC separation | -80°C/LN2 | ≥18 yr (iNTS+) cases/NCs/HCs | GH/BF (with site specific adjustments) |
|  | 8-10 mL | Immune assay | IVI/REF LAB | Centrifuge/PBMC separation | -80°C/LN2 | ˂18 yr (iNTS+) cases/NCs/HCs | GH/BF (with site specific adjustments) |
|  | 3-5 mL | Immune assay | IVI/REF LAB | Centrifuge | -80°C | All age group (iNTS+) cases/NCs/HCs | MD/ET/DRC/NI (with site specific adjustments) |
|  | 1 mL | HEP^15^ serology | IVI | Centrifugation | -80°C | Pregnant women ± Fever ± Jaundice | TBD |
| Surgical tissue | 5-10 mm^3^ | Tissue PCR^16^ | IVI/REF LAB | PBS^17^ buffer | -80°C | Inclusion criteria (if surgery required) | All |
|  |  | Tissue culture for isolation of Salmonella | Field sites | Manual culture | Store isolates |  |  |
|  |  | Tissue histopathology |  | H&E^18^ staining | Store stain slides |  |  |
| Peritoneal fluid | 10 mL | Culture for isolation of Salmonella |  | Automated culture system/Manual culture | Store isolate |  |  |
|  |  | PCR | IVI/REF LAB | Peritoneal fluid aliquot | -80°C |  |  |
| Stool | 5-10 gram | Stool culture | Field sites | Direct/Enrichment Culture/Sensitivity | Isolate -80°C | TF/PF/iNTS(+) cases/NCs/HCs | All |
|  | 1 g Aliquot | Immunological assay/molecular | IVI/REF LAB | N/A | -80°C | TF/PF/iNTS(+) cases/NCs/HCs | All |
|  |  | Microbiome analysis | IVI/REF LAB | DNA^19^ extraction on-site or at REF LAB | -80°C | TF/PF/iNTS(+) cases/NCs/HCs | All (if feasible) |
| OPS | 1 swab | Throat culture | Field sites | Direct/Enrichment Culture/Sensitivity | Isolate -80°C | iNTS(+) cases/NCs/HCs | All |
|  | 1 swab | PCR | IVI/REF LAB | N/A | -80°C | iNTS(+) cases/NCs/HCs | All |
|  | 1 swab | PCR for Group A *Streptococcus(*GAS) | IVI | N/A | -80°C | Inclusion criteria | All |
| Urine | 10 mL | Antibiotic metabolite testing | Field sites/  IVI/REF LAB | N/A | 1mL at -80°C | Inclusion criteria | All |

^1^ Storage at field depot: -80°C/LN2 (liquid nitrogen).

^2^ Applicable participants: blood culture positive typhoid fever [TF(+)], paratyphoid fever [PF(+)], and iNTS disease [iNTS(+)] cases; neighborhood controls (NCs); household contacts (HCs).

^3^ Applicable countries: All [Burkina Faso (BF), Democratic Republic of Congo (DRC), Ethiopia (ET), Ghana (GH), Madagascar (MD), Nigeria (NI)].

^4^ mL: milliliter. ^5^ NA: Not applicable. ^6^ b/w: body weight. ^7^ RDT: Rapid diagnostic test. ^8^ HIV: Human immunodeficiency virus. 9 TBD: To be decided. (iNTS positive cases in study sites in Ghana).

^10^ Rt-PCR: Reverse Transcription Polymerase Chain Reaction. ^11^ TBS (Trypticase Soy Broth)-Oxgall. ^12^ REF LAB: Reference laboratory. ^13^ PBMC: Peripheral blood mononuclear cell.

^14^ LN2: Liquid nitrogen tank. ^15^ HEP: Hepatitis. ^16^ PCR: Polymerase Chain Reaction. ^17^ PBS: Phosphate buffered saline. ^18^ H&E staining: Hematoxylin and eosin staining. ^19^ DNA: Deoxyribonucleic acid.
